# Unresolved grief in women and men in Sweden three years after undergoing unsuccessful in vitro fertilization treatment

**DOI:** 10.3109/00016349.2010.512063

**Published:** 2010-09-17

**Authors:** Helena Volgsten, Agneta Skoog Svanberg, Pia Olsson

**Affiliations:** Department of Women's and Children's Health, Uppsala University, Uppsala, Sweden

**Keywords:** Depression, grief, infertility, unsuccessful IVF, qualitative interviews

## Abstract

*Objective*. To explore the experience of undergoing unsuccessful in vitro fertilization (IVF) treatment and of remaining childless 3 years after IVF in both women and men. *Design*. Qualitative-approach study. *Sample*. Ten women and nine men who had attended a public fertility clinic in Sweden. *Methods*. Individual qualitative semi-structured interviews were conducted with qualitative content analysis guiding the analysis. *Results*. Three years after the end of IVF treatment, most men and women were still processing and had not adapted to childlessness, indicating that the grieving process was unresolved. Unsuccessful IVF was experienced by women in terms of grief, whereas men took upon themselves a supportive role and did not express grief. A need for professional support and counseling in how to handle grief was described. An unstructured end after IVF treatment left unanswered questions. *Conclusions*. The grieving process after unsuccessful IVF treatment was hampered among both men and women. The provision of additional individual support during IVF is recommended as men and women experienced childlessness differently. Support and counseling concerning grief reactions following IVF failure, and a structured final consultation after IVF may facilitate the grieving process after undergoing unsuccessful IVF treatment.

## Introduction

Infertility is conceptualized as a major crisis in life. A crisis evokes emotional reactions that are classified into four main phases: the initial phase (shock, surprise, denial); the reactive phase (frustration, anger, anxiety, guilt, grief, depression, isolation); the adaptive phase (acceptance) and a resolution phase (planning for future solutions) (1,2). Reactions during a crisis are determined by factors such as: influences of the event itself, pre-existing personality, cultural factors and social support from close family and friends (3). The conceptual framework of crisis theory (2,4) is modified for the purpose of the infertility crisis (1). Although the crisis theory may initially be used, this theory is considered less useful for the adaptive phase as infertility may consist of recurring stressful events (5). Women who have undergone unsuccessful tubal surgery for female infertility factor mostly remain in the reactive phase for 2 years after surgery (6). Life crises are considered time-limited with duration of 6 weeks or less for the reactive phase (1). The infertility crisis may be ongoing or chronic after recurrent treatment failure.

Infertility represents many losses, such as, the loss of fertility and reproductive ability and the loss of a child and biological offspring (1). Thus, grief is a normal reaction to a distressing situation, such as a loss (7). However, when the loss is of a potential, not an actual loss, the couple may not realize they are allowed to grieve (1). Grief reactions are often self-limited and with preserved self-esteem (8). The duration of normal grief reactions depends on the grieving process, which is considered important for successful adjustment (1,7). However, the grieving process may be hampered or prolonged, thus causing complicated grief. Complicated grief occurs when grief reactions persist more than 2 months after a loss and is consistent with the definition of major depression (9). Therefore, distinguishing between grief reactions, complicated grief and depression is important but can be difficult (8,10). Symptoms that are not characteristic for normal grief reactions are excessive guilt, suicidal ideation and feelings of worthlessness (9).

Approximately 30–40% of couples undergoing IVF treatment will remain childless after treatment (11,12). Grief is one of the main experiences of being childless in women 2 years after ending unsuccessful IVF (13). Previous qualitative studies exploring the experience of childlessness after undergoing unsuccessful IVF have focused on women (13–15) or interviews have been conducted with the couple together, not individually (16,17). Hence, there is a need to obtain a more in-depth understanding about both men and women's experiences of remaining childless after undergoing unsuccessful IVF treatment.

The objective of the current qualitative-approach study was to explore the experience of undergoing unsuccessful IVF treatment and of remaining childless 3 years after IVF in a sample of women and men in Sweden.

## Material and methods

Men and women, who had undergone IVF or ICSI (intra-cytoplasmic sperm injection) treatment at the Centre of Reproduction, University Hospital, Uppsala, Sweden, participated. In Sweden, assisted reproductive technology (ART) is offered by both public and private clinics. The Centre of Reproduction is a public clinic and infertile couples are offered three subsidized IVF treatments with a waiting list of 3 months at the time of the study. Subsidized counseling was offered to all couples at their first visit to the public clinic.

The methodological approach was qualitative with semi-structured individual interviews (18) and qualitative content analysis (19). Participants were identified from the clinic's database and recruitment was in two steps. First, an invitation letter including a written consent to participate was sent to women (*n* = 49) who had undergone IVF and had no further IVF treatments at the public clinic after a minimum of 3 years. Secondly, men and women were contacted individually by phone, by the first author (HV) about a week after the letter was received; this provided an opportunity for the potential participants to ask questions, to check for exclusion criteria unavailable in the database and to book a place and time for an interview. Reasons for declining participation in the study were not assessed. Exclusion criteria were: Swedish language difficulties, having a biological child from previous relationship and having already adopted a child.

All interviews were conducted in a non-clinical room at the hospital in 2006 by the first author. Written informed consent was obtained and socio-demographic and fertility data were collected via a questionnaire. Fertility history, such as, on a previous pregnancy loss, was obtained from the medical records. The interviews started after informing the participant that she or he could withdraw participation at any time.

A pre-tested and revised interview-guide covering the following topics was used: life situation as involuntary childless; mental health during and 3 years after IVF; partner relationship and social network and support; and decision-making when to end IVF treatment. All interviews were tape-recorded, lasted about 20–60 minutes (mean 40 minutes) and were then transcribed verbatim by the first author. Field notes on the interaction during the interview were made and taken into consideration during the analysis. The interviewer was known to some participants due to working as a midwife at the Centre of Reproduction.

### Data analyses

All interviews were analyzed using qualitative content analysis (19) by the first author and checked against the co-authors. After careful reading of the transcribed interview, the text was divided into meaning units. A meaning unit is a piece of text with a specific content that relates to the aim of the study. All meaning units were then condensed; a process of shortening the text while still preserving the core content, into condensed meaning units. The condensed meaning units were further shortened into codes; a labeling that allows the data to be understood in relation to the context. Codes were then grouped into categories depending on similarities and differences in content (19).

The study was approved by the Ethics Committee, Uppsala University, Uppsala, Sweden.

## Results

The study group consisted of 19 participants: 10 women and 9 men. Seven were couples and two male and three female participants did not bring their partners to the study. Of the latter participants, two of the men and two of the women had a biological child with partners from a previous relationship and these partners were excluded. One female participant was divorced. Three of the male and two of the female participants were planning for adoption at the time of the interview. Demographic and fertility information are presented in Table 1. The participants had their last IVF treatment at the public clinic at a mean of 38 months prior to the data collection. The analyses resulted in two main categories and seven sub-categories describing the experiences of the participants (see Table 2). Quotations from the interviews are presented below and labeled by M (male) or F (female), code number and age.

**Table 1:** Demographic and fertility data given by women and men.

	Women (*n* = 10)	Men (*n* = 9)
Age (years)	39.6 (35–43)	38.8 (36–46)
University/college (*n*)	3	4
High school	7	5
Employee (*n*)	9	7
Unemployed	1	0
Student	0	2
Duration of infertility (years)^a^	7.0 (2–10)	4.7 (1–7)
Infertility factors^b^ (*n*)		
Female	2	4
Male	4	1
Unexplained/not known	4	4
Previous pregnancy loss (*n*, %)		
No	4 (40%)	
Yes	6 (60%)	
Numbers of IVF/ICSI^b^ (*n*)		
Public	2.3 (1–3)	2.1 (1–3)
Numbers of IVF/ICSI^b^ (*n*)	3	2
Private	3.0 (2–4)	2.5 (2–3)
Counseling (*n*)		
During IVF	5	1
After IVF	0	0

a Time before last IVF.

b Data from data base.

**Table 2:** Men's and women's experiences after undergoing unsuccessful IVF treatment

Experiences in relation to unsuccessful IVF treatment
Putting up a shield
Late realization of the need of professional support
Affected partner relationship
Frustrated at the ending of IVF
Experiences of remaining childless after IVF failure
Unanswered questions after ending IVF
Feeling excluded and lacking understanding
Loss of future life goals

### Putting up a shield

Unsuccessful IVF affected mental health negatively. One male reflection was that not everyone undergoing IVF was mentally strong enough to deal with failure after failure. Women explained how they had been putting up a shield of optimism prior to treatment and not showing any reactions after failure. Undergoing unsuccessful IVF treatment was described by women in terms of grief. The experience after treatment failure was described as losing someone close, and suicidal thoughts were revealed. Men had no knowledge on possible emotional reactions after unsuccessful treatment, and the reactions of their wives were unexpected. Not allowing oneself to grieve, to ignore grief and just continue with the next treatment were described by both men and women.

during these five years, after each try it was just… new attempt … we never allowed ourselves to grieve (…) we ignored it and went on to the next attempt … and that was also the same with those around us … *(F0537)*.

### Late realization of the need of professional support

A lack of professional support for handling the grief reactions after IVF failure was described by both men and women. They did not request support during treatment and not until IVF treatment ended did they realize that professional support would have been beneficial. Men felt it was expected of them to take the supportive role toward their partner and show no sadness or grief when treatment failed. Handling their own and their partners’ grief reactions after treatment failure were considered difficult. In retrospect, a lack of knowledge about grief might have hindered their partners from proper grieving, according to some men.

it was more a support role I took on myself as I thought this was required (…) but to allow grief… allowed us both to be sad … instead of … yes, now she is sad so I have to comfort her and keep myself together a bit longer … then I can't be sad at the same time … so you kept yourself together and swept it under the mat (…) at the same time I hinder her from grieving properly *(M0436)*.

Individual support was suggested by men as this would enable them to better understand their spouses' unexpected grief reactions. Mandatory counseling after IVF failure, to able to process grief in between the treatments, was suggested by both men and women. Men expressed that their partner would have needed professional support both during and after treatment.

### Affected partner relationship

Partner relations were affected both positively and negatively by unsuccessful IVF. Some men and women described how the relationship with their partner had been strengthened in a critical situation. Other men and women experienced strain in the relationship and temporary separations after ending IVF. Difficulty in communicating was one reason mentioned by women who experienced strain in the relationship. Another reason was a lack of self-esteem, expressed as worthlessness, upon not being a proper woman.

when I felt so bad … there was something wrong with me I wasn't a proper woman, I didn't understand why my husband wanted to stay with me … because I couldn't give him what he wanted … I felt worthless … I thought I couldn't do anything … *(F0741)*.

The subject of sexuality was spontaneously mentioned during the interview by some women but not by men. Sexual problems were a reason for temporary separation after IVF. The lust and enjoyment of sexual life disappeared during IVF treatment and had not returned.

### Frustrated at the ending of IVF

During the treatment neither men nor women were given advice about whether to end or continue further IVF treatments. Men considered receiving information about the prognosis of undergoing further IVF would have been helpful in their decision-making. The reasons for discontinuing IVF treatment included the women's emotional reactions, medical and economical factors. Men explained that it was their wife's decision not to continue further IVF treatment and they did not always agree with their spouse's decision, and frustration over the decision was expressed. The women themselves stated that it had been their decision to discontinue IVF. After the last IVF treatment, a final consultation with a health professional was lacking according to both men and women. Treatments were described as being too forced and the ending often being abrupt. Both men and women were frustrated at being left on their own when treatment failed.

… finished … so quick … that was the end, it was finished … there was also this … someone to talk to afterwards is needed … so it doesn't become as detached as it feels today … it went so fast … *(M0337)*.

### Unanswered questions after ending IVF

Three years after IVF there were still unanswered questions. Women were concerned that they had not been thoroughly evaluated, not being fully examined or not having the fertility factor finally explained were the reasons given by both men and women as to why it was difficult to process childlessness. Both men and women described that an unexplained fertility factor was difficult to accept and it was difficult to explain the cause of infertility to others. Men considered an explained infertility factor was easier to deal with as it could be taken care of. A female factor for infertility was described by women as hard to process, as the husband then could have a child with another woman. However, an explained factor was more difficult for the one carrying the factor according to both men and women.

yes, I think that means a lot … I think that is so for the person who has something wrong with them … that person … I have been there … I think that he can have children … with another woman … you don't just go out and have a child with whoever … I think that it is a grief for those that can't … *(F0835)*.

### Feeling excluded and lacking understanding

For men, childlessness meant being left out in social situations when friends and colleagues were talking about their children; men felt excluded.

… to be involuntarily … childless … means some form of exclusion … unless you have … I experience that it is something you can't share with many others … on a different level … it can be ordinary things … such as if you have friends that talk about their children and so on … so I would probably see that as some kind of exclusion … *(M0937)*.

Women described childlessness as not being like others, with inability to have children and create a family. Both men and women had experienced upsetting questions and a lack of understanding from their family members, friends and colleagues. Men expressed that being childless affected contact with their own parents, as there were no grandchildren connecting them. The lack of understanding was explained as lack of knowledge about infertility and its treatment, and a disinterest in what it meant to be childless. Women expressed feelings of frustration and anger on the lack of understanding. Men and women communicated differently about their fertility problems. Men described how they seldom informed their closest family, friends and colleagues about the fertility problems, and as a consequence their social network avoided confronting them about their childlessness. Women described more often how they communicated with the family and friends, who consequently tried to support them, but women experienced loss of privacy by telling colleagues at work.

### Loss of future life goals

Three years after ending IVF mental health was described as relatively stable among men. Women revealed being both more mentally stable than during IVF but some were still suffering from grief; it was difficult not knowing how to handle grief. Pain and the absence of a child were the experiences of childlessness described among men. Women described the experience in terms of grief, emptiness, and meaninglessness.

… grief … it is a deep grief … yes, that is perhaps the strongest word that comes directly … emptiness … yes, the meaningfulness … there is something missing in your life … in any case when you reach this age … you feel … what is life about … it maybe that … the child part so to say, … it feels as if you have been cheated … *(F0939)*.

Most men and women had not reached an adaptation to childlessness. Men described how they tried to learn to live with the pain by denying it. Men were uncertain whether their spouse had accepted childlessness as the fertility problem was not discussed at home. Women described how they were physically and rationally close to accepting childlessness; however, there would never be an emotional acceptance. Both men and women described that having children was something they had considered self-evident. Blaming oneself for being childless and feelings of guilt were expressed by women, but not by men. The women felt a loss of control over the situation because they were childless. The loss of parenthood, of not having a family including a child, was described as a different life situation; but at the same time, a life situation with more freedom. Some women still had hopes for spontaneous pregnancy but also expressed how the hope for a child had taken a great deal of time in their life. This was one reason for ending the process, but they were still grieving about being childless. The future, as described by men and women, was being alone in old age. Women explained that there would be no one to succeed them and thought about what to do with their inheritance. Both men and women described disappointment at not being able to give their parents the joy of grandchildren and not being able to have their own grandchildren.

## Discussion

Three years after ending IVF treatment most men and women were still processing and had not adapted to remaining childless, indicating the grieving process was unresolved. Unsuccessful treatment was experienced by women in terms of grief, whereas men took upon themselves a supportive role and did not express grief reactions, but expressed a need for professional counseling in how to handle grief.

The results from the current study support the statement that the crisis theory is insufficient for infertile couples (5), as men and women were still processing and had not adapted to childlessness. Men and women tend to describe emotional reactions according to typical gender roles (16). This was corroborated by the differences in experiences between men and women, such as men having the supportive role, and was in accordance with a study of women and men reacting differently 2 years after unsuccessful tubal surgery (6). Women in the current study described the experience of undergoing unsuccessful treatment in terms of grief, in accordance with previous studies (13–15). Furthermore, women in the current study also described symptoms of depression after IVF failure: as a lack of self-esteem, expressed as worthlessness. A loss of control and blaming oneself for being childless and feelings of guilt were also expressed by women, but not by men. Depression includes feelings of worthlessness, such as loss of self-esteem and is contrary to normal grief reactions where self-esteem is not affected (8). Guilt and loss of control, such as sense of control, are considered as vulnerability factors for distress after IVF (20). There are two aspects of loss of control: of bodily functions and of future life goals (21). Following multiple pregnancy losses and failed treatments, women may be particularly vulnerable to chronic grief (22). Previous pregnancy loss was an independent risk factor for major depression in women in our previous study (23) and had occurred among more than half of the women in the current study. Furthermore, pre-existing personality is considered a determinant factor (3) and women with neurotic personality may be more vulnerable to develop depression after treatment failure (24). Relational strain among men and women has also been shown to be a significant determinant for developing depressive symptoms after failed treatment (25). For some couples, this was one of the reasons for another loss, which included temporary separations or divorce. More losses due to infertility and fewer gains of childlessness, were reported in this study than in previous studies reporting more gains (13,17).

A lack of professional support and how men took the supportive role after treatment failure were also described and indicated that men were in need of their own individual support and were in need of their own individual support while undergoing IVF treatment. Although assisted reproduction has been developed during the last two decades, little seems to have improved in terms of support and counseling for couples undergoing IVF, a situation described by Schmidt a decade ago (26). Suggestions for improvement for those providing IVF were given by men in this study which included individual and mandatory support, including information about possible grief reactions and how to handle them, as there was no prior awareness of the need for professional support after failed treatment. These are clinical implications that are possible for a specialized health professional in the IVF team, such as the midwife, to assess for all couples undergoing IVF. Such improvements are needed to facilitate the grieving process after undergoing unsuccessful IVF treatment.

One consequence of grief and depressive symptoms after IVF failure was to discontinue treatment. The decision to end treatment was described by men as being the woman's decision, one reason being grief reactions after treatment failure. The most common reason for discontinuing treatment is emotional burden (27,28). Thus, to limit dropout from further treatment, more effective support may allow the couple an opportunity to reflect upon their options undergoing treatment. Furthermore, to have follow-up appointments, already booked prior to IVF, could be another option for counseling and support when treatment has failed. If depressive symptoms are identified and adequately treated, the chance may be that more couples continue and optimize the chance of successful outcome after treatment (28).

Lack of a structured end to the IVF treatment may be a factor hampering the processing of childlessness. However, there are no rituals, when ending IVF, for the experience of ongoing childlessness to facilitate an adaptation (1,5). To have some kind of structured summary after IVF, as a final consultation, is important to process the experience of involuntary childlessness. Improved consultation is also suggested to facilitate the decision-making process for the couple on when to end IVF (29). However, ending treatment most often means that the possibilities for having a biological child are over and includes giving up hope of pregnancy. This was expressed by women in the current study by being rationally close to accepting childlessness but not emotionally. Therefore, cessation of treatment does not end infertility for women, as there is no emotional acceptance, indicating increased vulnerability to develop depression (30).

Unanswered questions 3 years after IVF were considered as interfering with the processing of childlessness and previous studies indicate that an unexplained infertility factor can be a distress factor hampering the processing of childlessness (14,31) and may lead to depression in men (23). Explanation of the infertility diagnosis, at a final consultation may increase the sense of control (6), facilitate the processing of childlessness and decrease the risk of developing depression. Undoubtedly, a final consultation would be beneficial for couples in resolving remaining issues. With a shorter waiting time for infertility evaluation and treatment and fewer visits to the clinic than a decade ago (26), there is less time and opportunity for explaining the cause of infertility either before or during IVF. Therefore, the result of the evaluation, whether an explained or unexplained factor has been found, needs to be thoroughly clarified to the couple not only during IVF but also at a final consultation. Information about other treatment options or alternatives available or whether to end treatment needs to be discussed. Counseling may be helpful for the couple in their decision-making process within this often ambiguous situation.

Social isolation, a feeling of being excluded with no understanding from their social network, was experienced by both men and women after IVF, and communication about fertility problems with close family members and friends differed between men and women, resulting in less social support among men. A lack of social support or discontent with support given leads to more grief and depression in women (32). For those who actively seek social support and express negative feelings, a healthier mental health outcome is described than for those who deny negative feelings and do not seek or accept support from their social network (3).

A loss of future life goals was described among men and women in the current study who had not adapted to remaining childless 3 years after undergoing IVF. Another study revealed that among women who consider themselves as definitely childless, substantial proportion have high scores of complicated grief and depressive symptoms (32). It is important for health professionals to distinguish between normal grief and complicated grief (10) and identify and follow the individuals at risk of developing depression after IVF failure and to offer evidence-based treatment. However, among some women there was still a hope for spontaneous pregnancy and among other participants there were early plans for adoption, indicating that they were not planning to remain childless.

Qualitative research aims to attain meaning and understanding of individual's experience (19,33). The qualitative design of this study enabled assessment of the experiences of infertile individuals in more detail and depth compared to a quantitative approach using questionnaires. One concern with assessment by questionnaires is that self-report measures may be susceptible to social desirability bias. Infertile couples may want to present as well-adjusted emotional state especially prior to treatment (34) and may respond more positively in questionnaires (29). Qualitative research interviews with its interactive characteristics have a potential to diminish this tendency. The interviews were all conducted, transcribed and analyzed by the same person, who had been working as a midwife at the clinic where the participants previously had undergone IVF. This provided an initial insight and knowledge in the research field, a pre-understanding considered important in qualitative research. However, when interpreting the results, on the other hand, it is important to clarify the pre-understanding (19,33). Therefore, to problematize the interpretation, the co-author who had not worked in an IVF-setting discussed alternative interpretations if considered necessary. However, transferability of these results has to be made with caution. The extent of the transferability of the result of a qualitative study to other settings and populations depends on cultural and traditional similarities or differences (18).

A limitation of the present study is that men with male infertility factor and women with university level education were few compared to those attending the clinic where recruitment was done (35). Furthermore, individuals with other ethnic backgrounds than Swedish are not included. Another limitation was that sexual problems, spontaneously mentioned by women, were not a topic guiding the interviews and therefore the male participants' experiences of sexuality was not assessed.

In conclusion, unresolved grief was the main experience of remaining childless after undergoing unsuccessful IVF. The recommendations for reproductive health professionals are to provide additional individual support, as men and women were experiencing involuntary childlessness differently. Information and counseling in an early phase of IVF, concerning grief reactions following IVF failure is recommended. A structured final consultation after IVF, with time to discuss the result of the infertility evaluation and if other treatment alternatives are available, may facilitate the grieving process after undergoing unsuccessful IVF treatment.
